# Association of Patient Demographic Characteristics and Insurance Status With Survival in Cancer Randomized Clinical Trials With Positive Findings

**DOI:** 10.1001/jamanetworkopen.2020.3842

**Published:** 2020-04-30

**Authors:** Joseph M. Unger, Charles D. Blanke, Michael LeBlanc, William E. Barlow, Riha Vaidya, Scott D. Ramsey, Dawn L. Hershman

**Affiliations:** 1 SWOG Cancer Research Network Statistics and Data Management Center, Seattle, Washington; 2 Fred Hutchinson Cancer Research Center, Seattle, Washington; 3 SWOG Cancer Research Network Group Chair’s Office, Knight Cancer Institute, Oregon Health & Science University, Portland; 4 Columbia University Medical Center, New York, New York

## Abstract

**Importance:**

Few new treatments tested in phase 3 cancer randomized clinical trials show an overall survival benefit. Although understanding whether the benefits are consistent among all patient groups is critical for informing guideline care, individual trials are designed to assess the benefits of experimental treatments among all patients and are too small to reliably determine whether treatment benefits apply to demographic or insurance subgroups.

**Objective:**

To systematically examine whether positive treatment effects in cancer randomized clinical trials apply to specific demographic or insurance subgroups.

**Design, Setting, and Participants:**

Cohort study of pooled patient-level data from 10 804 patients in SWOG Cancer Research Network clinical treatment trials reported from 1985 onward with superior overall survival for those receiving experimental treatment. Patients were enrolled from 1984 to 2012. Maximum follow-up was 5 years.

**Main Outcomes and Measures:**

Interaction tests were used to assess whether hazard ratios (HRs) for death comparing standard group vs experimental group treatments were associated with age (≥65 vs <65 years), race/ethnicity (minority vs nonminority populations), sex, or insurance status among patients younger than 65 years (Medicaid or no insurance vs private insurance) in multivariable Cox regression frailty models. Progression- or relapse-free survival was also examined. Data analyses were conducted from August 2019 to February 2020.

**Results:**

In total, 19 trials including 10 804 patients were identified that reported superior overall survival for patients randomized to experimental treatment. Patients were predominantly younger than 65 years (67.3%) and female (66.3%); 11.4% were black patients, and 5.7% were Hispanic patients. There was evidence of added survival benefits associated with receipt of experimental therapy for all groups except for patients with Medicaid or no insurance (HR, 1.23; 95% CI, 0.97-1.56; *P* = .09) compared with those with private insurance (HR, 1.66; 95% CI, 1.44-1.92; *P* < .001; *P* = .03 for interaction). Receipt of experimental treatment was associated with reduced added overall survival benefits in patients 65 years or older (HR, 1.21; 95% CI, 1.11-1.32; *P* < .001) compared with patients younger than 65 years (HR, 1.41; 95% CI, 1.30-1.53; *P* < .001; *P* = .01 for interaction), although both older and younger patients appeared to strongly benefit from receipt of experimental treatment. The progression- or relapse-free survival HRs did not differ by age, sex, or race/ethnicity but differed between patients with Medicaid or no insurance (HR, 1.32; 95% CI, 1.06-1.64; *P* = .01) vs private insurance (HR, 1.74; 95% CI, 1.54-1.97; *P* < .001; *P* = .03 for interaction).

**Conclusions and Relevance:**

Patients with Medicaid or no insurance may have smaller added benefits from experimental therapies compared with standard treatments in clinical trials. A better understanding of the quality of survivorship care that patients with suboptimal insurance receive, including supportive care and posttreatment care, could help establish how external factors may affect outcomes for these patients.

## Key Points

**Question:**

Do positive treatment effects from cancer clinical trials apply to all sociodemographic groups?

**Findings:**

In this cohort study using patient-level data recorded for 10 804 patients who participated in cancer randomized clinical trials with positive findings, the receipt of experimental treatment vs standard treatment was associated with improved overall survival among patients 65 years or older compared with patients younger than 65 years. No significant added benefit of experimental treatment was observed among patients having Medicaid or no insurance compared with those with private insurance.

**Meaning:**

Patients with Medicaid or no insurance may have smaller added benefits from experimental therapies in clinical trials.

## Teaser

This cohort study uses pooled patient-level data from the SWOG Cancer Research Network to assess whether positive treatment effects are associated with demographic or insurance subgroups among patients participating in phase 3 cancer randomized clinical trials.

## Introduction

Phase 3 trials are designed to evaluate the efficacy of new treatments or interventions. Trials with positive results—especially those with a survival end point—often establish new standards of care.^1^ However, phase 3 trials are typically designed to examine their primary end point for all patients combined and lack statistical power to show that the benefit of the experimental treatment consistently applies to major demographic, socioeconomic, and clinical subgroups.^2,3^ In oncology, this is critical to determine because treatment benefits could be smaller in disadvantaged groups at potentially higher risk of noncancer-related deaths and with more limited access to health care resources. Numerous prior studies have described the reduced health care utilization rates and cancer outcomes for racial/ethnic minority populations and for patients with Medicaid or no insurance (M/NI).^4,5,6,7,8,9,10,11^ In addition, differences in survival by age and sex for many cancers have been clearly characterized.^12^ Yet the generalizability of the treatment effects to these subgroups is often assumed, and few, if any, distinctions within demographic or socioeconomic subgroups are made in cancer treatment guidelines.

Approximately 1 in 4 phase 3 cancer clinical trials shows a benefit, and fewer show improved overall survival (OS).^13,14^ Using more than 3 decades of clinical trials conducted in the SWOG Cancer Research Network,^15^ we systematically examined whether the treatment effects for trials with positive findings applied to important demographic and insurance subgroups.

## Methods

Clinical trials are conducted by SWOG for patients with a broad range of cancer types. In the present cohort study, we identified SWOG phase 3 clinical treatment trials, completed in 1985 or later, for which a significant benefit for OS in favor of the new experimental therapy was reported in the primary published article (eTable 1 in the Supplement). Patient-level data from these positive trials were pooled. Patients were enrolled from 1984 to 2012. The present study followed the Strengthening the Reporting of Observational Studies in Epidemiology (STROBE) reporting guideline.^16^ Each trial in this secondary analysis was previously approved by an institutional review board; written informed consent was previously obtained from all patients. No one received compensation or was offered any incentive for participating in this study.

### Study Populations

Patients with missing age, sex, race, or performance status were excluded. Ethnicity data were routinely collected in SWOG trials beginning in 1991, and insurance status in 1992. By design, the analysis examining the association of sex with treatment outcomes was restricted to non–sex-specific cancers, the analysis by minority racial/ethnic populations restricted to patients with complete data on race and ethnicity, and the analysis by insurance type restricted to patients younger than 65 years because patients 65 years of age or older are covered by Medicare.

### Variables and Adjustment Covariates

We defined key independent variables of interest, including age (≥65 vs <65 years), sex, and any minority racial/ethnic population (black, Hispanic, Asian, Native American, or Pacific Islander vs white; by self-report). Insurance status was categorized as private vs M/NI based on prior studies.^6,7^

To account for differences in clinical risk that may be associated with survival across a diverse set of cancers, a baseline clinical health status variable was used based on performance status, which has been strongly associated with cancer outcomes and was available for all studies.^17^ Performance status was dichotomized as 0 (no functional limitation) vs 1 or higher (some functional limitation) because few patients (7%) had a performance status of 2 or higher and several studies excluded such patients (eTable 1 in the Supplement). In addition, a global (study-level) disease stage variable was created, which was defined as advanced or poor prognosis or locally advanced disease vs other (eTable 1 in the Supplement).

### Insurance Analysis by Follow-up Time and Sensitivity Analysis

We also examined the extent to which insurance status was associated with the effect of treatment on both OS and progression- or relapse-free survival (PFS) over time by iteratively truncating follow-up at 0.1-year intervals through 7.5 years. The robustness of the interaction analyses for all variables was examined by iteratively excluding each individual study and evaluating the statistical strength of the association among remaining studies by using the regression model Wald χ^2^ statistic.

### Statistical Analysis

The primary end point was OS given the clinical relevance and a definition consistent across different cancer and treatment settings. It was defined as the time from randomization until death due to any cause. Patients known to be alive at the last contact date were censored. Progression- or relapse-free survival was also examined with cancer-specific definitions.

To emphasize the association of cancer treatment with outcomes, the primary analyses for each factor used survival within the first 5 years after randomization. We used interaction tests to examine whether the association of treatment with survival differed by demographic and insurance status variables. Multivariable Cox regression frailty models were used with each individual study considered a random effect to account for heterogeneity in design, assessment intervals, follow-up duration, and prognosis.^18,19^ We included covariate adjustment for age, sex, minority status, period of registration (during or before 2000 vs after 2000), clinical risk using performance status, and the study-level disease stage variable.

Statistical tests were from Wald χ^2^ test statistics derived from multivariable Cox regression frailty models. Analyses were conducted from August 2019 to February 2020 using SAS, version 9.4 (SAS Institute Inc) and R, version 3.4.1 (R Foundation for Statistical Computing). All tests were 2-sided, and *P* < .05 was considered statistically significant.

## Results

**Figure 1.**
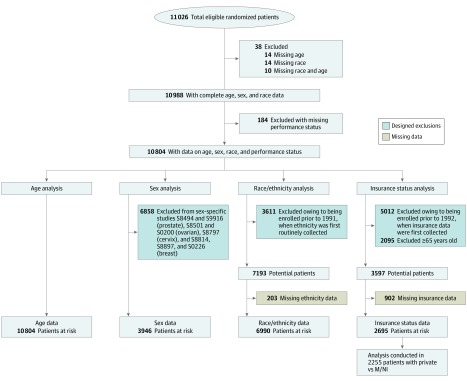
Study Population for Each Sociodemographic Variable Analysis M/NI represents Medicaid or no insurance.

Nineteen phase 3 treatment trials showing an overall survival benefit were identified across a diverse range of cancers (eTable 1 in the Supplement).^20,21,22,23,24,25,26,27,28,29,30,31,32,33,34,35,36,37,38^ In all cases, the experimental therapy was compared with an existing standard of care that included active treatment. Of 11 026 eligible patients, 38 had missing data on age or race, and 184 had missing data on performance status, leaving 10 804 patients (98.0%) with complete data on age, sex, race, and performance status (Figure 1). The age analysis was conducted for the entire cohort of 10 804 patients. Eight trials with 6858 patients with sex-specific cancers were excluded from the analysis by sex, leaving 3946 patients. Overall, 3611 patients were enrolled prior to 1991, leaving 7193 patients with race/ethnicity data potentially analyzable for the race/ethnicity analysis; of these patients, 203 (2.8%) had missing data on ethnicity, leaving 6990 patients. Overall, 5792 patients were enrolled in 1992 or after, among whom 2095 were 65 years or older, leaving 3597 patients with potentially analyzable insurance data; of these, 902 patients (25.1%) had missing insurance data, leaving 2695 patients. The insurance analysis was conducted in 2255 patients with private insurance or M/NI and included 16 trials with at least partial insurance data and 10 trials with nearly complete (>85%) insurance data (eTable 1 in the Supplement).

### Characteristics of Patients

The patients were predominantly younger than 65 years (67.3%), 66.3% were female, and 35.7% had a performance status of 1 or higher (eTable 2 in the Supplement). In the non–sex-specific cancer trials only, 37.6% were female, consistent with the US cancer population.^39^ Of the patients with complete data on race/ethnicity, 20.5% were in a minority population, including 11.4% black and 5.7% Hispanic patients. Of the patients included in the insurance analysis, 24.8% had M/NI.

In the age analysis, older patients were less likely to be in a minority population or be fully active (Table 1). In the sex analysis, female patients were less likely to have advanced disease. In the race/ethnicity analysis, minority patients were less likely to be older than 65 years or to be fully active. In the insurance analysis, patients with M/NI were more commonly minority patients and less likely to be fully active. There was no evidence that the association between age, sex, race/ethnicity, or insurance status and demographic or clinical status variables differed between those assigned to standard and those assigned experimental therapy.

**Table 1.** Characteristics of Patients by Factor Level and Treatment Group

Characteristic	By factor level, No. (%) of patients		*P* value^a^	By treatment group and factor level, No. (%) of patients				*P* value^a^^,^^b^
				Standard group		Experimental group		
**Age analysis (n = 10 804)**	**<65 y**	**≥65 y**		**<65 y**	**≥65 y**	**<65 y**	**≥65 y**	
Patients, No. (%)	7268 (67.3)	3536 (32.7)		3334 (67.5)	1607 (32.5)	3934 (67.1)	1929 (32.9)	
Sex								
Female	5318 (73.2)	1825 (51.6)	.20	2402 (72.0)	768 (47.8)	2916 (74.1)	1057 (54.8)	.34
Male	1950 (26.8)	1711 (48.4)		932 (28.0)	839 (52.2)	1018 (25.9)	872 (45.2)	
Race/ethnicity								
Minority	1434 (19.7)	518 (14.6)	<.001	630 (18.9)	234 (14.6)	804 (20.4)	284 (14.7)	.89
Not minority	5834 (80.3)	3018 (85.4)		2704 (81.1)	1373 (85.4)	3130 (79.6)	1645 (85.3)	
PS								
Fully active	5090 (70.0)	1852 (52.4)	.002	2317 (69.5)	792 (49.3)	2773 (70.5)	1060 (55.0)	.23
<Fully active	2178 (30.0)	1684 (47.6)		1017 (30.5)	815 (50.7)	1161 (29.5)	869 (45.0)	
Stage group								
Advanced	2716 (37.4)	2226 (63.0)	.06	1320 (39.6)	1064 (66.2)	1396 (35.5)	1162 (60.2)	.20
Not advanced	4552 (62.6)	1310 (37.0)		2014 (60.4)	543 (33.8)	2538 (64.5)	767 (39.8)	
**Sex analysis (n = 3946)**^c^	**Female**	**Male**		**Female**	**Male**	**Female**	**Male**	
Patients, No. (%)	1483 (37.6)	2463 (62.4)		706 (37.6)	1171 (62.4)	777 (37.6)	1292 (62.4)	
Age, y								
<65	926 (62.4)	1579 (64.1)	.15	448 (63.5)	741 (63.3)	478 (61.5)	838 (64.9)	.36
≥65	557 (37.6)	884 (35.9)		258 (36.5)	430 (36.7)	299 (38.5)	454 (35.1)	
Race/ethnicity								
Minority	302 (20.4)	494 (20.1)	.20	137 (19.4)	229 (19.6)	165 (21.2)	265 (20.5)	.69
Not minority	1181 (79.6)	1969 (79.9)		569 (80.6)	942 (80.4)	612 (78.8)	1027 (79.5)	
PS								
Fully active	752 (50.7)	1238 (50.3)	.78	360 (51.0)	597 (51.0)	392 (50.5)	641 (49.6)	.46
<Fully active	731 (49.3)	1225 (49.7)		346 (49.0)	574 (49.0)	385 (49.5)	651 (50.4)	
Stage group								
Advanced	826 (55.7)	1638 (66.5)	.02	378 (53.5)	759 (64.8)	448 (57.7)	879 (68.0)	.82
Not advanced	657 (44.3)	825 (33.5)		328 (46.5)	412 (35.2)	329 (42.3)	413 (32.0)	
**Race/ethnicity analysis (n = 6990)**^d^	**Minority**	**Not Minority**		**Minority**	**Not Minority**	**Minority**	**Not Minority**	
Patients, No. (%)	1431 (20.5)	5559 (79.5)		637 (20.0)	2541 (80.0)	794 (20.8)	3018 (79.2)	
Age, y								
<65	1084 (75.8)	3626 (65.2)	<.001	483 (75.8)	1651 (65.0)	601 (75.7)	1975 (65.4)	.66
≥65	347 (24.2)	1933 (34.8)		154 (24.2)	890 (35.0)	193 (24.3)	1043 (34.6)	
Sex								
Female	882 (61.6)	3821 (68.7)	.51	369 (57.9)	1688 (66.4)	513 (64.6)	2133 (70.7)	.09
Male	549 (38.4)	1738 (31.3)		268 (42.1)	853 (33.6)	281 (35.4)	885 (29.3)	
PS								
Fully active	835 (58.4)	3646 (65.6)	.007	351 (55.1)	1595 (62.8)	484 (61.0)	2051 (68.0)	.72
<Fully active	596 (41.6)	1913 (34.4)		286 (44.9)	946 (37.2)	310 (39.0)	967 (32.0)	
Stage group								
Advanced	714 (49.9)	2579 (46.4)	.82	347 (54.5)	1283 (50.5)	367 (46.2)	1296 (42.9)	.31
Not advanced	717 (50.1)	2980 (53.6)		290 (45.5)	1258 (49.5)	427 (53.8)	1722 (57.1)	
**Insurance analysis (n = 2255)**^e^	**M/NI**	**Private**		**M/NI**	**Private**	**M/NI**	**Private**	
Patients, No. (%)	558 (24.7)	1697 (75.3)		240 (23.1)	798 (76.9)	318 (26.1)	899 (73.9)	
Sex								
Female	347 (62.2)	1025 (60.4)	.70	152 (63.3)	459 (57.5)	195 (61.3)	566 (63.0)	.23
Male	211 (37.8)	672 (39.6)		88 (36.7)	339 (42.5)	123 (38.7)	333 (37.0)	
Race/ethnicity								
Minority	297 (53.2)	305 (18.0)	<.001	134 (55.8)	140 (17.5)	163 (51.3)	165 (18.4)	.17
Not minority	261 (46.8)	1392 (82.0)		106 (44.2)	658 (82.5)	155 (48.7)	734 (81.6)	
PS								
Fully active	272 (48.7)	1017 (59.9)	<.001	106 (44.2)	452 (56.6)	166 (52.2)	565 (62.8)	.71
<Fully active	286 (51.3)	680 (40.1)		134 (55.8)	346 (43.4)	152 (47.8)	334 (37.2)	
Stage group								
Advanced	335 (60.0)	1000 (58.9)	.86	157 (65.4)	503 (63.0)	178 (56.0)	497 (55.3)	.68
Not advanced	223 (40.0)	697 (41.1)		83 (34.6)	295 (37.0)	140 (44.0)	402 (44.7)	

Abbreviations: M/NI, Medicaid or no insurance; PS, performance status.

^a^ Determined from logistic regression accounting for study-level heterogeneity by specifying study as a random effect.

^b^ Represents whether the association between the factor and the category of interest differs between those receiving standard or experimental therapy, using an interaction test.

^c^ Analysis limited to patients enrolled to trials in non–sex-specific cancers only.

^d^ Analysis limited to patients with complete data on race/ethnicity.

^e^ Analysis limited to patients younger than 65 with private insurance or M/NI.

### Overall Survival Treatment Interactions

**Figure 2.**
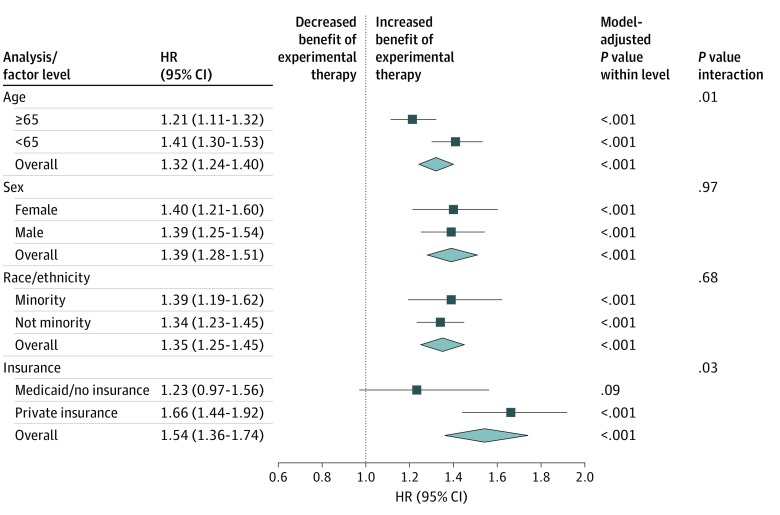
Association of Treatment With Overall Survival by Level of Demographic and Insurance Variables Forest plot showing the hazard ratio (HR) of death for patients receiving standard arm vs experimental arm therapies. Boxes represent HRs; horizontal lines, 95% CIs; diamonds, overall average HR across subgroups; and diamond size, 95% CI. The vertical line is the line of equal hazard (ie, neither an increased or decreased benefit of experimental therapy). Results for each sociodemographic variable level are derived from a single-adjusted model controlling for the covariates specified in the Methods.

Study-level estimates of the interaction between treatment and sociodemographic variables are shown in eFigure 1 in the Supplement. For the age analysis, receipt of experimental treatment was associated with reduced added OS benefits in patients 65 years or older (hazard ratio [HR], 1.21; 95% CI, 1.11-1.32; *P* < .001) compared with patients younger than 65 (HR, 1.41; 95% CI, 1.30-1.53; *P* < .001; *P* = .01 for interaction), although the receipt of experimental therapy was strongly associated with added OS benefits for both age groups (Figure 2; eTable 3 in the Supplement).

Among patients in trials for non–sex-specific cancers, there was no evidence that the HR for standard to experimental therapy differed for female (HR, 1.40; 95% CI, 1.21-1.60; *P* < .001) vs male (HR, 1.39; 95% CI, 1.25-1.54; *P* < .001) patients (*P* = .97 for interaction). Among patients in the race/ethnicity analysis, receipt of experimental therapy appeared to be associated with increased added OS benefits in minority patients (HR, 1.39; 95% CI, 1.19-1.62; *P* < .001) compared with nonminority patients (HR, 1.34; 95% CI, 1.23-1.45; *P* < .001), although the difference between groups was small and not statistically significant (*P* = .68 for interaction).

For patients with M/NI, there was no statistically significant added benefit associated with receipt of experimental treatment (HR, 1.23; 95% CI, 0.97-1.56; *P* = .09) compared with patients with private insurance (HR, 1.66; 95% CI, 1.44-1.92; *P* < .001; *P* = .03 for interaction). Alternatively, although there was no difference in risk of death between patients with M/NI and privately insured patients treated with standard therapies (HR, 1.03; 95% CI, 0.84-1.27; *P* = .77), among patients receiving experimental therapies, those with M/NI had a greater risk of death; in other words, they did not have the same added benefits of experimental therapy (HR, 1.40; 95% CI, 1.14-1.71; *P* = .001) (eTable 3 in the Supplement).

### PFS Treatment

There was no evidence that patterns of association of treatment with PFS differed among subgroups by age, sex, or race/ethnicity (Table 2; eTable 4 in the Supplement). Among patients with private insurance, there was an observed greater benefit associated with receipt of experimental therapy (HR, 1.74; 95% CI, 1.54-1.97; *P* < .001) compared with patients with M/NI (HR, 1.32; 95% CI, 1.06-1.64; *P* = .01; *P* = .03 for interaction).

**Table 2.** Association of Treatment With Progression- or Relapse-Free Survival by Level of Demographic and Insurance Variables^a^

Factor	Hazard ratio (95% CI)	*P* value	
		Null	Interaction
Age, y			
≥65	1.33 (1.23-1.44)	<.001	.18
<65	1.43 (1.33-1.53)	<.001	
Overall	1.38 (1.31-1.46)	<.001	
Sex			
Female	1.43 (1.26-1.62)	<.001	.53
Male	1.51 (1.37-1.66)	<.001	
Overall	1.48 (1.37-1.60)	<.001	
Race/ethnicity			
Minority	1.33 (1.16-1.53)	<.001	.15
Not minority	1.49 (1.39-1.60)	<.001	
Overall	1.43 (1.33-1.53)	<.001	
Insurance status			
Medicaid or no insurance	1.32 (1.06-1.64)	.01	.03
Private insurance	1.74 (1.54-1.97)	<.001	
Overall	1.63 (1.46-1.81)	<.001	

^a^ Results by sociodemographic variable–level are derived from a single-adjusted model controlling for the covariates specified in the Methods section.

### Insurance Analysis by Follow-up Time

**Figure 3.**
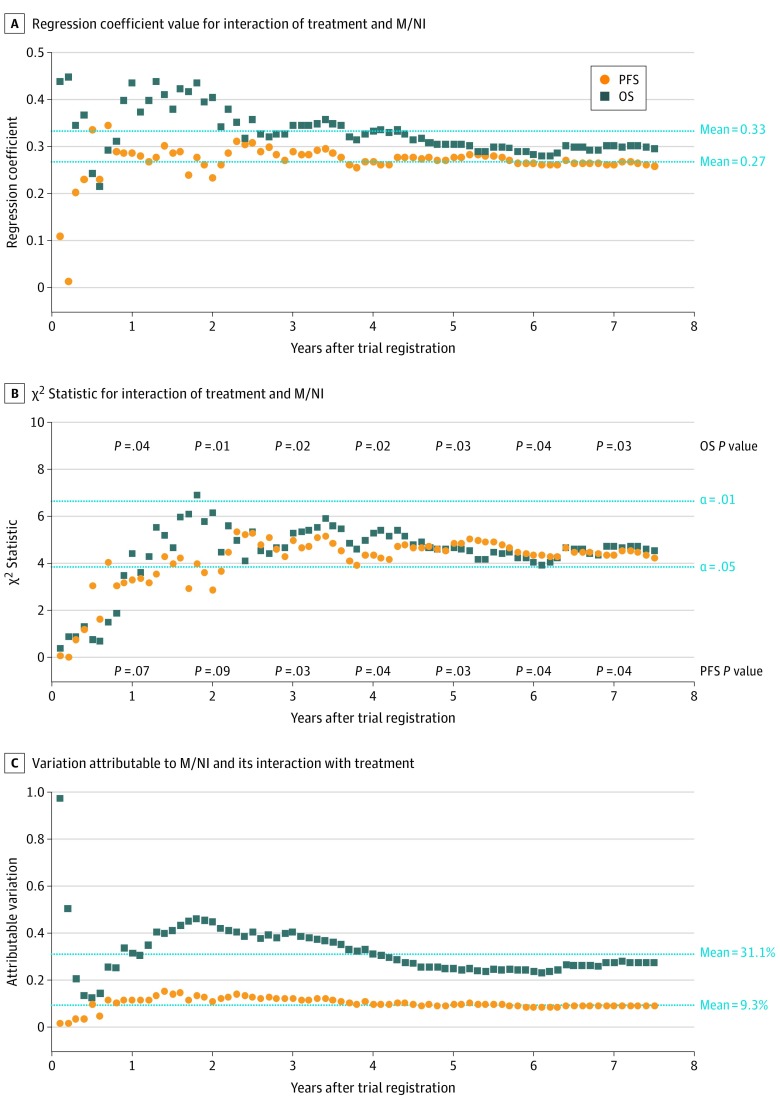
Association Between Insurance Status, Treatment, and Outcomes by Amount of Follow-up The regression coefficient (A), χ^2^ statistic (B), and attributable variation (C) for the interaction between insurance status and treatment depending on the amount of follow-up are shown. The strength of the interaction of treatment and insurance status was insensitive to the designated amount of follow-up time if specified annually, with interaction *P* values for the association with overall survival (OS) and with progression- or relapse-free survival (PFS) shown (B). M/NI represents Medicaid or no insurance.

The magnitude of the model coefficient value for the interaction of treatment and insurance status was approximately constant for both OS and PFS from 1 year after randomization onward (Figure 3A). Furthermore, the strength of the interaction for both OS and PFS reached statistical significance by 1 year and was insensitive to the designated amount of follow-up time when specified annually (Figure 3B). When considering only the model effect of treatment, insurance, and the interaction between insurance and treatment, nearly one-third of the variation in OS (31.1%)—but only 9.3% of the variation in PFS—was attributable to insurance status and its interaction with treatment (Figure 3C). This finding suggested that, even though insurance status was associated with both cancer and noncancer outcomes, its association with noncancer outcomes was notably greater. These results indicated a marked association of insurance status with both cancer and especially noncancer outcomes manifesting early in the course of treatment and remaining approximately constant through 7.5 years.

### Sensitivity Analysis

The findings were largely robust to the exclusion of individual studies (eFigure 2 in the Supplement). The χ^2^ statistic remained statistically significant in 15 of 19 cases (78.9%) for the interaction of age and treatment and in 12 of 16 cases (75.0%) for the interaction of insurance and treatment. The interaction tests were not statistically significant for the sex and race/ethnicity analyses in all cases.

## Discussion

In this pooled analysis of phase 3 studies positive for OS, receipt of experimental treatment was not associated with added OS benefits among patients with M/NI, in an adjusted model compared with privately insured patients. Furthermore, an association of insurance status with both PFS and OS was observed within 1 year after initiation of treatment and was sustained through 7.5 years. By contrast, significant added OS benefits from receipt of experimental therapies were observed for female patients, male patients, minority patients, nonminority patients, and privately insured patients. The magnitude of the added survival benefit associated with receiving experimental therapies was significantly greater for patients younger than 65 years based on the interaction analysis; however, receipt of experimental therapy was strongly associated with added OS benefits for both age groups, suggesting a quantitative rather than qualitative interaction, with more limited clinical or policy implications.^40^ Finally, the findings by insurance status, age, sex, and race/ethnicity were generally not influenced by the inclusion of any single trial, suggesting that these findings were internally robust.

Ayanian et al^41^ was among the first to identify that patients with M/NI had worse survival outcomes than other patients and concluded that differences in access to care, staging, and treatments played a role. Noncancer-related factors also likely play a role.^42^ Our study differs from prior studies in 2 key aspects. First, prior studies focused on differences between those with suboptimal insurance compared with those with private insurance overall, irrespective of the nature of the treatment (eg, newly proven treatment vs standard treatment).^42,43,44,45,46^ Second, in contrast to prior studies that relied on cancer population data, patients in clinical trials are uniformly staged according to eligibility criteria, a principal advantage of using trial data to examine outcomes by demographic and insurance factors. This uniform staging limits the potential for differences between patient groups in baseline health status (eg, differences in patterns of concurrent illnesses) to bias the association with outcomes. In addition, we adjusted for important baseline factors, including performance status, a key measure of health status and a potential confounder. Nonetheless, residual differences in health status and health behaviors between certain groups may remain, especially for disadvantaged groups. In addition, once enrolled, trial patients all have access to protocol-guided therapy representing either standard treatment or an experimental alternative thought to be approximately as good or better than standard of care. Thus, patients in clinical trials start with the same treatments, are healthy enough to participate in trials, and typically have similar initial cancer risk profiles, although differences in comorbid conditions not ruled out by protocol eligibility criteria may still exist.

An individual’s health care insurance facilitates their access to health care services; insurance status also reflects socioeconomic status.^47,48^ These 2 constructs are related because insurance coverage is associated with income.^47^ Medicaid eligibility is largely limited to children, parents of dependent children, pregnant women, disabled persons, and elderly persons and is primarily determined by income level; in this context, it can be considered a surrogate for poverty or low income, even as the program provides needed access to health care services.^48,49^ Importantly, the present study did not indicate that patients with M/NI did not benefit from cancer therapy. Rather, it showed that the *added* benefits associated with experimental therapies in clinical trials on OS were much smaller for patients with M/NI compared with privately insured patients. Indeed, within the group of patients with M/NI, the CI did not preclude some modest positive association with OS. The patients with M/NI did have improved PFS, suggesting that receipt of experimental therapies was associated with benefits for cancer-specific outcomes despite these benefits being much lower compared with privately insured patients.

The reasons for these findings are likely multifactorial. The observation that patients with M/NI had limited benefits associated with receipt of experimental therapies that were otherwise beneficial for all demographic patient groups is consistent with the idea that lower socioeconomic status has a constant, negative influence on health through the mechanism of long-term, sustained lack of access to resources.^50^ This could dampen the benefits of experimental treatments in trials for patients with suboptimal insurance if receipt of experimental therapy typically requires more supportive services or is more difficult to adhere to than standard care. Alternatively, access to postprotocol therapy may differ for those receiving experimental vs standard therapy. Under these scenarios, the added benefits of new experimental therapies may be short-lived. In fact, further detailed analyses in the present study showed that the association of insurance status with outcomes began within 1 year after treatment initiation, illustrating how the benefits of equal access to initial treatment quickly waned for patients with M/NI. Other reasons for the observed findings may include that patients with suboptimal insurance are likely at increased risk of noncancer deaths, which can reduce the ability to detect the benefits of experimental treatments in trials. This factor may also explain the more limited benefits of the experimental therapies observed among older patients because the burden of other (comorbid) diseases increases markedly with age.^51^

Legislation sponsored by the American Society of Clinical Oncology and currently before Congress seeks to require state Medicaid programs to cover routine patient costs for items and services that are provided in connection with a qualifying clinical trial regarding cancer or other life-threatening conditions.^52^ This legislation could help resolve the disparity in experimental treatment benefits by insurance status within clinical trials; however, this legislation would only improve the applicability of trial results if Medicaid patients receiving cancer treatment outside of trials also have adequate access to cancer care. Yet state-level Medicaid programs vary widely in their coverage and reimbursement policies, which may affect both the consistency and the adequacy of care for patients.^53,54^ One study highlighted how Medicaid coverage for hematopoietic cell transplant to treat patients with hematologic cancers varied substantially by state, with no states providing full recommended coverage.^55^ Variations in state Medicaid policies may be associated with time to receipt of surgery for breast cancer.^56^ Studies have shown underuse of screening, genetic testing and counseling, adjuvant therapy, surgery, and radiation therapy for patients on Medicaid.^57,58,59,60,61,62^ Furthermore, not all physicians accept Medicaid patients given the relatively low reimbursement rates, presenting additional hurdles to access to care for these patients.^63^ In general, our findings highlight the importance of coverage expansion policies for those with suboptimal insurance and underline the importance of improved coverage provisions for those with existing Medicaid insurance.

### Strengths and Limitations

To our knowledge, this is first study to examine whether treatment effects from cancer randomized clinical trials with positive findings were associated with major demographic or insurance status subgroups. This analysis required the unique combination of a large clinical trial database with patient-level data from multiple trials positive for OS conducted over a long period, with insurance status data collected for the same trials. Despite its uniqueness, the study also has limitations. Although performance status is strongly associated with outcomes irrespective of cancer type,^64^ its use as a covariate may not adequately reflect baseline health status. Insurance status data were not available for older trials. In addition, most of the trials were completed before the implementation of the insurance exchanges and the Medicaid expansion through the Affordable Care Act; thus, the potential influence of this legislation on the present findings could not be established. Given no known prior systematic examinations about how treatment effects may differ by sociodemographic variables, we reported *P* < .05 as statistically significant with no adjustment for multiple comparisons. Thus, confirmatory analyses in independent studies may provide important insights about the observed associations in this study.

## Conclusions

The magnitude of added treatment benefits associated with receipt of experimental therapies may not be uniform for patients with Medicaid or without insurance. Future work into the potential causes of this phenomenon is warranted. A better understanding of the quality of survivorship care that patients with suboptimal insurance receive, including supportive care and posttreatment care, could help establish how external factors may affect outcomes for these patients.

## Notes

Supplement. **eTable 1.** SWOG Studies With Statistically Significant Benefit of Experimental Therapy on Overall Survival **eTable 2.** Characteristics of the Study Sample **eFigure 1.** Study Level Estimates of the Interaction Between Treatment and Sociodemographic Variables With Respect to Overall Survival **eTable 3.** Association Between Treatment and Socioeconomic Variables With Respect to Overall Survival **eTable 4.** Association Between Treatment and Socioeconomic Variables With Respect to Progression-Free Survival **eFigure 2.** Interaction *P* Values By Factor; Results Excluding Single Studies **eReferences.**
