# Supplementary material for: Association of Patient Demographic Characteristics and Insurance Status With Survival in Cancer Randomized Clinical Trials With Positive Findings
Source: JAMA Netw Open. 2020 Apr 30;3(4):e203842. doi: 10.1001/jamanetworkopen.2020.3842 (PMC7193331; doi:10.1001/jamanetworkopen.2020.3842)
Supplement: Supplement. — eTable 1. SWOG Studies With Statistically Significant Benefit of Experimental Therapy on Overall Survival eTable 2. Characteristics of the Study Sample eFigure 1. Study Level Estimates of the Interaction Between Treatment and Sociodemographic Variables With Respect to Overall Survival eTable 3. Association Between Treatment and Socioeconomic Variables With Respect to Overall Survival eTable 4. Association Between Treatment and Socioeconomic Variables With Respect to Progression-Free Survival eFigure 2. Interaction P Values By Factor; Results Excluding Single Studies eReferences. [file jamanetwopen-3-e203842-s001.pdf]

Supplementary Online Content

Unger JM, Blanke CD, LeBlanc M, et al. Association of patient demographic characteristics and insurance status with survival in cancer randomized clinical trials with positive findings. *JAMA Netw Open*. 2020;3(4):e203842. doi:10.1001/jamanetworkopen.2020.3842

eTable 1. SWOG Studies With Statistically Significant Benefit of Experimental Therapy on Overall Survival

eTable 2. Characteristics of the Study Sample

eFigure 1. Study Level Estimates of the Interaction Between Treatment and Sociodemographic Variables With Respect to Overall Survival

eTable 3. Association Between Treatment and Socioeconomic Variables With Respect to Overall Survival

eTable 4. Association Between Treatment and Socioeconomic Variables With Respect to Progression-Free Survival

eFigure 2. Interaction *P* Values By Factor; Results Excluding Single Studies

eReferences.

This supplementary material has been provided by the authors to give readers additional information about their work.

© 2020 Unger JM et al. *JAMA Network Open*.

eTable 1. SWOG Studies With Statistically Significant Benefit of Experimental Therapy on Overall Survival

Study number, cancer type	Major Eligibility Criteria [ca stage category, 1 v 2][a]	Treatment Comparison[b] Experimental therapy vs. Standard therapy	Accrual Years	Database Year	Reported HR (p-value)	N[c]	Insurance data (%)
S8494[1], Prostate	Stage D2; PS=0-3 [1]	**Leuprolide plus flutamide** vs. leuprolide	1985-1986	2019	1.30 (p=.035)[d]	515	0.0%
S8501[2], Ovarian	Previously untreated; stage III; PS=0-2 [1]	**Intraperitoneal cisplatin + cyclophosphamide** vs. intravenous cisplatin + cyclophosphamide	1986-1992	2002	1.32 (p=.02)	530	2.9%
S8591[3], Colon	Duke's stage C; PS=0-2 [2]	**5-FU + Levamisole vs.** observation	1984-1987	2019	1.49 (p=.006)	606	0.0%
S8624[4], Multiple Myeloma	Previously untreated; PS=0-3 [1]	**Chemotherapy plus dose intensive corticosteroids** vs. chemotherapy plus standard dose corticosteroids	1987-1990	1999	1.31[d](p=.02)	508	0.0%
S8710[5], Bladder	Transitional cell; stages T2–T4A; PS=0-1 [1]	**M-VAC plus cystectomy** vs. cystectomy alone	1988-1997	2002	1.33 (p=.06)[e]	255	43.1%
S8736[6], NHL	Inter. or high grade; stage I-IIE, non-bulky disease; PS=0-2 [2]	**CHOP plus radiotherapy** vs. CHOP alone	1988-1995	2000	1.70 (p=.02)	401	34.9%
S8797[7], Cervix	Stages IA2, IB, or IIA; PS=0-2 [2]	**Cisplatin/5-FU plus radiation therapy** vs. radiation therapy alone	1990-1996	1999	1.95 (p=.01)[f]	242	87.2%
S8814[1], Breast	T1-T3; postmenopausal; node positive, N1 or N2; PS not given [2]	**CAF followed by tamoxifen or CAF with concurrent tamoxifen** vs. tamoxifen alone	1989-1995	2002	1.20 (p=.04)	1463	48.4%
S8892[9], Head and neck	Nasopharyngeal; stages III-IV(M0); PS=0-2 [1]	**Cisplatin/5-FU plus radiation therapy** vs. radiation therapy alone	1989-1996	2001	2.50 (p=.005)	148	66.2%
S8897[10], Breast	T1-T3a, node negative; PS not given [2]	**CAF** vs. CMF	1989-1993	1999	1.19 (p=.06)[f]	2675	13.5%
S8949[11], Renal	Metastatic; PS=0-1 [1]	**Nephrectomy plus interferon alfa-2b** vs. interferon alfa-2b alone	1991-1998	2003	1.30 (p=.05)[d]	241	87.6%
S9008[12], Gastric	Stages IB–IV (M0); prior en bloc surgery; PS=0-2 [1]	**Surgery plus 5-FU/leucovorin/radiation therapy** vs. surgery alone	1991-1998	2005	1.35 (p=.005)	555	94.1%
S9126[13], AML	Refractory/relapse or secondary; PS=0-1 [1]	**Ara C-DNR plus CsA** vs. Ara C-DNR alone	1993-1998	1999	1.28 (p=.05)	222	100.0%
S9210[14], Multiple Myeloma	Previously untreated; PS not given [1]	**Prednisone 50 mg** vs. prednisone 10 mg (for remission maintenance)	1993-1998	2001	1.60 (p=.05)[d]	123	100.0%
S9308[15], NSCLC	Stages IIIB or IV; PS=0-1 [1]	**Cisplatin plus vinorelbine** vs. vinorelbine alone	1993-1995	1998	1.38 (p=.002)[d]	412	100.0%
S9916[16], Prostate	Advanced (metastatic) refractory; PS=0-3 [1]	**Docetaxel plus estramustine** vs. mitoxantrone plus prednisone	1999-2003	2019	1.25 (p=.02)	683	100.0%
S0200[17], Ovarian	Recurrent, stage III or IV; PS=0-1 [1]	**Pegylated liposomal doxorubicin plus carboplatin** vs. carboplatin	2002-2004	2015	2.38 (p=.02)	59	100.0%
S0226[18], Breast	Hormone-receptor positive; metastatic, postmenopausal, no prior systemic therapy; PS=0-2 [1]	**Anastrozole and fulvestrant** vs. anastrozole alone	2004-2009	2015	1.23 (p=.05)	691	100.0%
S0777[19], Multiple Myeloma	Newly diagnosed; PS=0-3 [2]	**Lenalidomide + dexamethasone + bortezomib vs.** lenalidomide + dexamethasone	2008-2012	2016	1.41 (p=.025)	475	100.0%
**TOTAL: 19 trials**			**1984-2012**			**10,804**	

PS=performance status; HR=hazard ratio; OS=overall survival; CI=confidence interval; Inter=intermediate; NHL=non-Hodgkin's lymphoma; VAD=vincristine, doxorubicin and dexamethasone; M-VAC= methotrexate, vinblastine, doxorubicin and cisplatin; CHOP=cyclophosphamide, doxorubicin, vincristine, and prednisone; 5-FU=fluorouracil; Ara C = Cytarabine; DNR=daunorubicin; CsA=cyclosporine-A; NSCLC=non-small cell lung cancer; CAF=cyclophosphamide, doxorubicin, and 5-fluorouracil; CMF=cyclophosphamide, methotrexate, and fluorouracil; AML=Acute myeloid leukemia

[a] Cancer stage category, 1 = Advanced or poor prognosis locally advanced, 2 = other.
[b] Experimental therapy indicated first in bold type.
[c] Excluding patients with missing age, sex, or performance status.
[d] HR estimate not reported; estimate calculated from primary manuscript dataset.
[e] The study was considered positive even though the p-value for the overall survival comparison was marginally greater than .05, based on the totality of the examinations of the overall survival endpoint.
[f] Study reported one-sided results per design specifications; reported as 2-sided here for consistency with other studies.

© 2020 Unger JM et al. *JAMA Network Open*.

eTable 2. Characteristics of the Study Sample

	Standard Therapy Arms (n=4,941)	Experimental Therapy Arms (n=5,863)	Overall (n=10,804)
Age			
<65 years	3,334 (67.5%)	3,934 (67.1%)	7,268 (67.3%)
>=65 years	1,607 (32.5%)	1,929 (32.9%)	3,536 (32.7%)
Sex[a]			
- All patients			
Female	3,170 (64.2%)	3,973 (67.8%)	7,143 (66.1%)
Male	1,771 (35.8%)	1,890 (32.2%)	3,661 (33.9%)
- Non-sex specific cancers only (n=3,946)			
Female	706 (37.6%)	777 (37.6%)	1,483 (37.6%)
Male	1,171 (62.4%)	1,292 (62.4%)	2,463 (62.4%)
Minority race/ethnicity[b] (N=6,990)			
Black	364 (11.5%)	434 (11.4%)	798 (11.4%)
Asian	78 (2.5%)	97 (2.5%)	175 (2.5%)
Native American	11 (0.3%)	14 (0.4%)	26 (0.4%)
Pacific Islander	7 (0.2%)	8 (0.2%)	15 (0.2%)
Hispanic	164 (5.2%)	232 (6.1%)	396 (5.7%)
Any Minority	637 (20.0%)	794 (20.8%)	1,431 (20.5%)
White	2541 (80.0%)	3018 (79.2%)	5,559 (79.5%)
Method of Payment[c] (N=2,257)			
Private	798 (76.9%)	900 (73.8%)	1,698 (75.2%)
Medicaid	108 (10.4%)	135 (11.1%)	243 (10.8%)
No Insurance	132 (12.7%)	184 (15.1%)	316 (14.0%)
Baseline Health Status[a,d]			
Fully Active	3,109 (62.9%)	3,833 (65.4%)	6,942 (64.3%)
Some Functional Limitation	1,832 (37.1%)	2,030 (34.6%)	3,862 (35.7%)
Advanced or locally advanced disease[a]			
Yes	2,384 (48.2%)	2,558 (43.6%)	4,942 (45.7%)
No	2,557 (51.8%)	3,305 (56.4%)	5,862 (54.3%)
Enrollment Year			
≤2000	4,106 (83.1%)	5,009 (85.4%)	9,115 (84.4%)
>2000	835 (16.9%)	854 (14.6%)	1,689 (15.6%)

[a] Rates differ by arm, p<.05 by chi-square test.
[b] Among patients with known race and ethnicity. Minority race/ethnicity defined to include black, Asian, Native American, Pacific Islander, and Hispanic. Excluding multiple/unknown.
[c] Among patients <65 years enrolled 1992 or after.
[d] Based on performance status, 0 (Fully Active) vs. ≥1 (Some function limitation).

© 2020 Unger JM et al. *JAMA Network Open*.

eFigure 1. Study Level Estimates of the Interaction Between Treatment and Sociodemographic Variables With Respect to Overall Survival

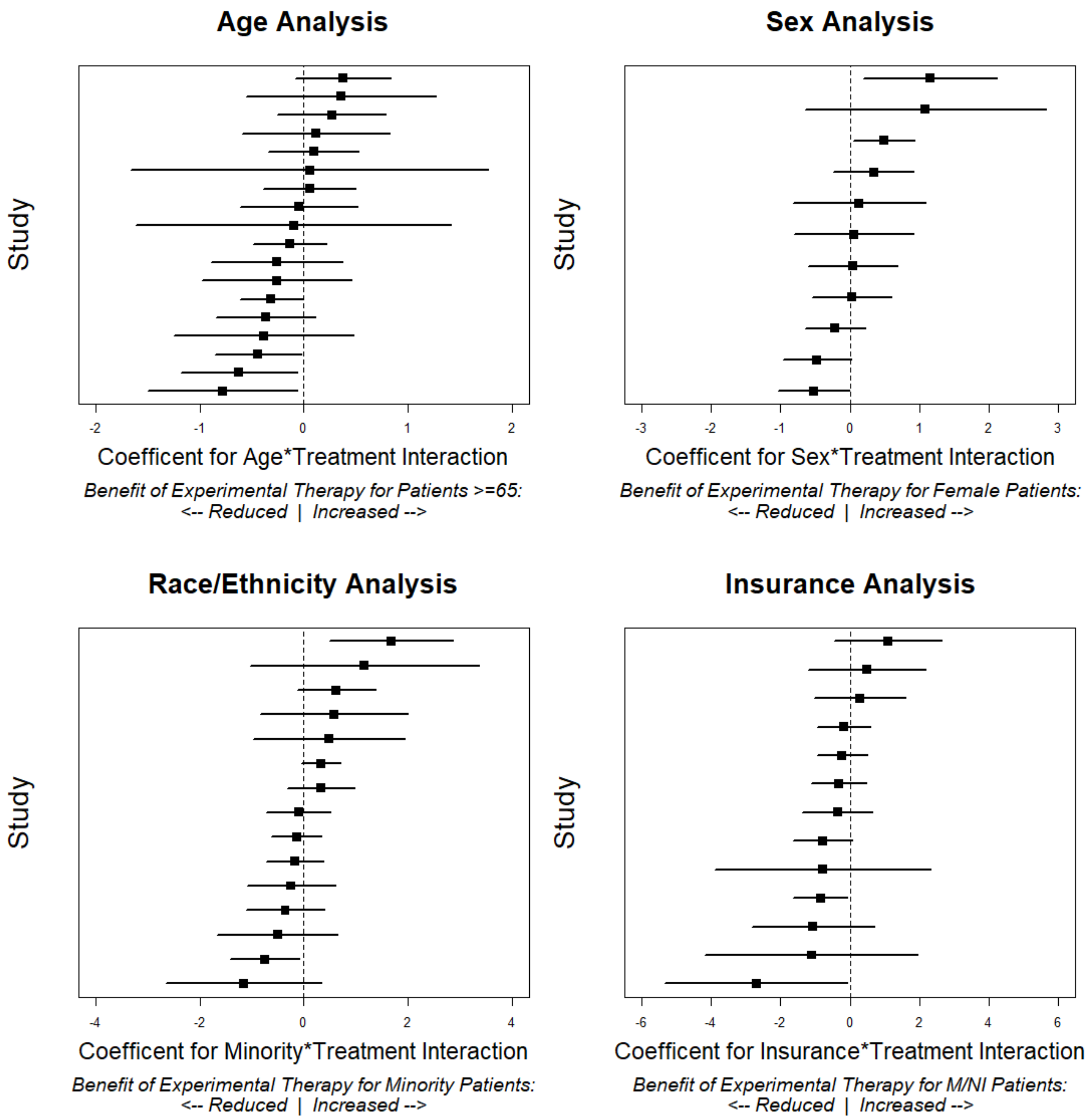

© 2020 Unger JM et al. *JAMA Network Open*.

eTable 3. Association Between Treatment and Socioeconomic Variables With Respect to Overall Survival[a]

	Treatment<br>HR (95% CI)<br>p-value		
	Standard Arm	Experimental Arm	HR (95% CI) for treatment within strata of factor
**Age Analysis**			
Age ≥65 years	1.57 (1.44-1.72)	1.30 (1.19-1.42)	1.21 (1.11-1.32)
	P<.001	P<.001	P<.001
Age <65 years	1.41 (1.30-1.53)	1.0	1.41 (1.30-1.53)
	P<.001		P<.001
HR (95% CI) for age w/in treatment strata	1.12 (1.02-1.22)	1.30 (1.19-1.42)	
	P=.01	P<.001	***Interaction p=.01***
**Sex Analysis**			
Female	1.29 (1.14-1.46)	0.93 (0.82-1.05)	1.40 (1.21-1.60)
	P<.001	P=.23	P<.001
Male	1.39 (1.25-1.54)	1.0	1.39 (1.25-1.54)
	P<.001		P<.001
HR (95% CI) for sex w/in treatment strata	0.93 (0.82-1.05)	0.93 (0.82-1.05)	
	P=.24	P=.23	***Interaction p=.97***
**Race/Ethnicity Analysis**			
Minority	1.45 (1.28-1.64)	1.05 (0.92-1.19)	1.39 (1.19-1.62)
	P<.001	P=.50	P<.001
Not minority	1.34 (1.23-1.45)	1.0	1.34 (1.23-1.45)
	P<.001		P<.001
HR (95% CI) for race/ethnicity w/in treatment strata	1.08 (0.96-1.23)	1.05 (0.92-1.19)	
	P=.20	P=.50	***Interaction p=.68***
**Insurance Analysis**			
Medicaid/No insurance	1.72 (1.39-2.12)	1.40 (1.14-1.71)	1.23 (0.97-1.56)
	P<.001	P=.001	P=.09
Private insurance	1.66 (1.44-1.92)	1.0	1.66 (1.44-1.92)
	P<.001		P<.001
HR (95% CI) for insurance w/in treatment strata	1.03 (0.84-1.27)	1.40 (1.14-1.71)	
	P=.77	P=.001	***Interaction p=.03***

[a] The results by sociodemographic variable level are derived from a single adjusted model controlling for the covariates specified in Methods.

© 2020 Unger JM et al. *JAMA Network Open*.

eTable 4. Association Between Treatment and Socioeconomic Variables With Respect to Progression-Free Survival[a]

	**Treatment** HR (95% CI) p-value		
	Standard Arm	Experimental Arm	HR (95% CI) for treatment within strata of factor
**Age Analysis**			
Age ≥65 years	1.45 (1.34-1.57)	1.09 (1.01-1.18)	1.33 (1.23-1.44)
	P<.001	P=.03	P<.001
Age <65 years	1.43 (1.33-1.53)	1.0	1.43 (1.33-1.53)
	P<.001		P<.001
HR (95% CI) for age w/in treatment strata	1.02 (0.94-1.10)	1.09 (1.01-1.18)	
	P=.69	P=.03	***Interaction p=.18***
**Sex Analysis**			
Female	1.44 (1.29-1.61)	1.01 (0.90-1.13)	1.43 (1.26-1.62)
	P<.001	P=.93	P<.001
Male	1.51 (1.37-1.66)	1.0	1.51 (1.37-1.66)
	P<.001		P<.001
HR (95% CI) for sex w/in treatment strata	0.96 (0.85-1.07)	1.01 (0.90-1.13)	
	P=.42	P=.93	***Interaction p=.53***
**Race/Ethnicity Analysis**			
Minority	1.43 (1.27-1.60)	1.07 (0.96-1.20)	1.33 (1.16-1.53)
	P<.001	P=.22	P<.001
Not minority	1.49 (1.39-1.60)	1.0	1.49 (1.39-1.60)
	P<.001		P<.001
HR (95% CI) for race/ethnicity w/in treatment strata	0.96 (0.85-1.07)	1.07 (0.96-1.20)	
	P=.43	P=.22	***Interaction p=.15***
**Insurance Analysis**			
Medicaid/No insurance	1.54 (1.27-1.86)	1.16 (0.97-1.39)	1.32 (1.06-1.64)
	P<.001	P=.10	P=.01
Private insurance	1.74 (1.54-1.97)	1.0	1.74 (1.54-1.97)
	P<.001		P<.001
HR (95% CI) for insurance w/in treatment strata	0.88 (0.73-1.06)	1.16 (0.97-1.39)	
	P=.18	P=.10	***Interaction p=.03***

[a] The results by sociodemographic variable level are derived from a single adjusted model controlling for the covariates specified in Methods.

© 2020 Unger JM et al. *JAMA Network Open*.

eFigure 2. Interaction *P* Values By Factor; Results Excluding Single Studies

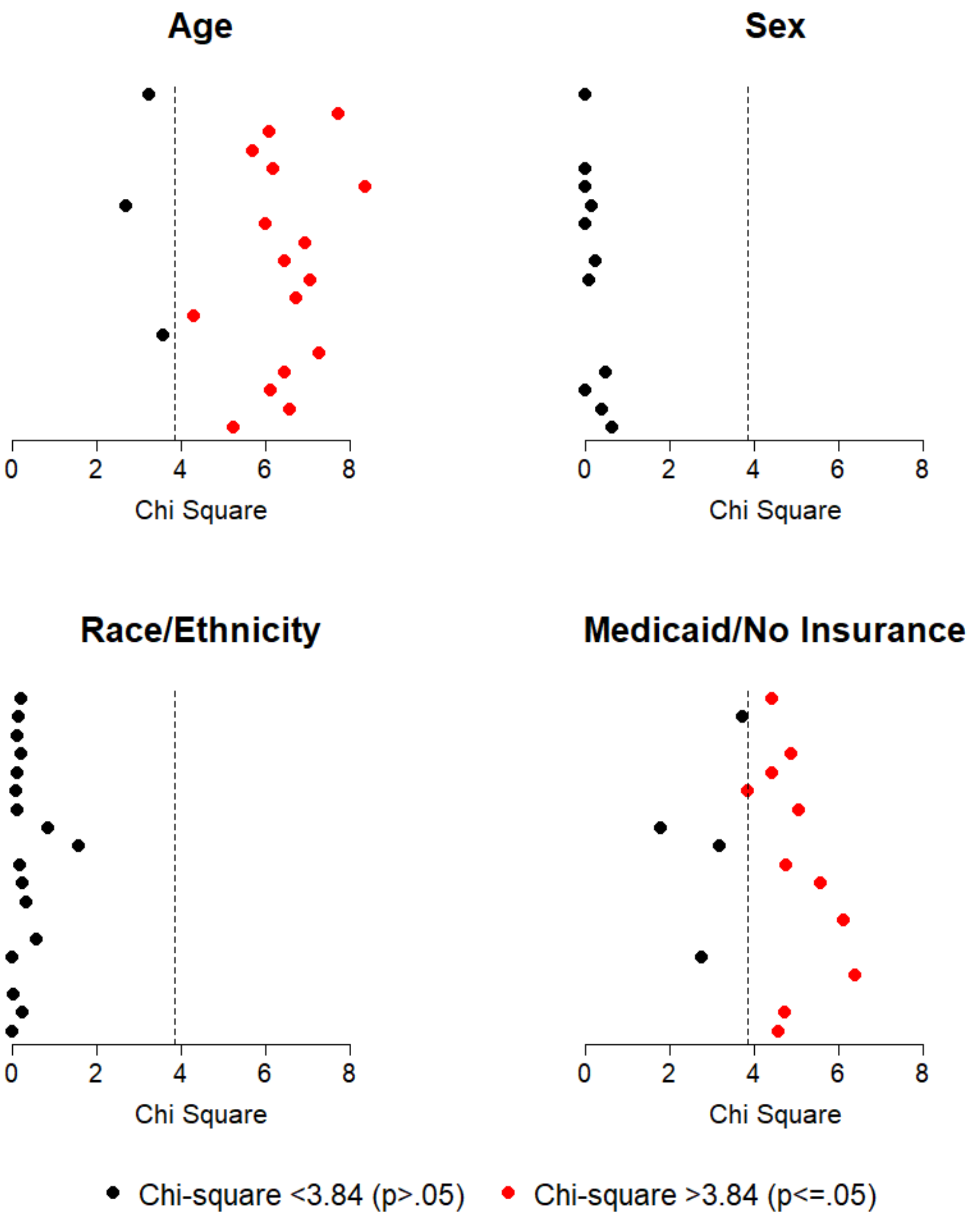

eFigure 1 indicates the chi-square test statistic for the interaction term for different factors. Each dot represents the analysis with one of the studies excluded. A blue dot indicates a statistically significant finding (.01<p<.05), a red dot indicates a highly statistically significant finding (p<.01), and a black dot indicates the finding was not statistically significant. In no case did the removal of any single study from the analysis results in statistically significant evidence that the average treatment effect differed between age, sex, or race/ethnicity subgroups (eFigure 1). In contrast, the findings by age and Medicaid/no insurance were generally consistent, regardless of whether any particular study was excluded. This cross-validation approach suggests indicates that the influence of no single study solely determined the results.

© 2020 Unger JM et al. *JAMA Network Open*.

eReferences

1. Crawford ED, Eisenberger MA, McLeod DG, et al: A controlled trial of leuprolide with and without flutamide in prostatic carcinoma. N Engl J Med 321:419-24, 1989
2. Alberts DS, Liu PY, Hannigan EV, et al: Intraperitoneal cisplatin plus intravenous cyclophosphamide versus intravenous cisplatin plus intravenous cyclophosphamide for stage III ovarian cancer. N Engl J Med 335:1950-5, 1996
3. Moertel CG, Fleming TR, Macdonald JS, et al: Fluorouracil plus levamisole as effective adjuvant therapy after resection of stage III colon carcinoma: a final report. Ann Intern Med 122:321-6, 1995
4. Barlogie B, Jagannath S, Vesole DH, et al: Superiority of tandem autologous transplantation over standard therapy for previously untreated multiple myeloma. Blood 89:789-93, 1997
5. Grossman HB, Natale RB, Tangen CM, et al: Neoadjuvant chemotherapy plus cystectomy compared with cystectomy alone for locally advanced bladder cancer. N Engl J Med 349:859-66, 2003
6. Miller TP, Dahlberg S, Cassady JR, et al: Chemotherapy alone compared with chemotherapy plus radiotherapy for localized intermediate- and high-grade non-Hodgkin's lymphoma. N Engl J Med 339:21-6, 1998
7. Peters WA, 3rd, Liu PY, Barrett RJ, 2nd, et al: Concurrent chemotherapy and pelvic radiation therapy compared with pelvic radiation therapy alone as adjuvant therapy after radical surgery in high-risk early-stage cancer of the cervix. J Clin Oncol 18:1606-13, 2000
8. Albain KS, Barlow WE, Ravdin PM, et al: Adjuvant chemotherapy and timing of tamoxifen in postmenopausal patients with endocrine-responsive, node-positive breast cancer: a phase 3, open-label, randomised controlled trial. Lancet 374:2055-2063, 2009
9. Al-Sarraf M, LeBlanc M, Giri PG, et al: Chemoradiotherapy versus radiotherapy in patients with advanced nasopharyngeal cancer: phase III randomized Intergroup study 0099. J Clin Oncol 16:1310-7, 1998
10. Hutchins LF, Green SJ, Ravdin PM, et al: Randomized, controlled trial of cyclophosphamide, methotrexate, and fluorouracil versus cyclophosphamide, doxorubicin, and fluorouracil with and without tamoxifen for high-risk, node-negative breast cancer: treatment results of Intergroup Protocol INT-0102. J Clin Oncol 23:8313-21, 2005
11. Flanigan RC, Salmon SE, Blumenstein BA, et al: Nephrectomy followed by interferon alfa-2b compared with interferon alfa-2b alone for metastatic renal-cell cancer. N Engl J Med 345:1655-9, 2001
12. Macdonald JS, Smalley SR, Benedetti J, et al: Chemoradiotherapy after surgery compared with surgery alone for adenocarcinoma of the stomach or gastroesophageal junction. N Engl J Med 345:725-30, 2001
13. List AF, Kopecky KJ, Willman CL, et al: Benefit of cyclosporine modulation of drug resistance in patients with poor-risk acute myeloid leukemia: a Southwest Oncology Group study. Blood 98:3212-20, 2001
14. Berenson JR, Crowley JJ, Grogan TM, et al: Maintenance therapy with alternate-day prednisone improves survival in multiple myeloma patients. Blood 99:3163-8, 2002
15. Wozniak AJ, Crowley JJ, Balcerzak SP, et al: Randomized trial comparing cisplatin with cisplatin plus vinorelbine in the treatment of advanced non-small-cell lung cancer: a Southwest Oncology Group study. J Clin Oncol 16:2459-65, 1998
16. Petrylak DP, Tangen CM, Hussain MH, et al: Docetaxel and estramustine compared with mitoxantrone and prednisone for advanced refractory prostate cancer. N Engl J Med 351:1513-20, 2004
17. Alberts DS, Liu PY, Wilczynski SP, et al: Randomized trial of pegylated liposomal doxorubicin (PLD) plus carboplatin versus carboplatin in platinum-sensitive (PS) patients with recurrent epithelial ovarian or peritoneal carcinoma after failure of initial platinum-based chemotherapy (Southwest Oncology Group Protocol S0200). Gynecol Oncol 108:90-4, 2008
18. Mehta RS, Barlow WE, Albain KS, et al: Combination anastrozole and fulvestrant in metastatic breast cancer. N Engl J Med 367:435-44, 2012
19. Durie BG, Hoering A, Abidi MH, et al: Bortezomib with lenalidomide and dexamethasone versus lenalidomide and dexamethasone alone in patients with newly diagnosed myeloma without intent for immediate autologous stem-cell transplant (SWOG S0777): a randomised, open-label, phase 3 trial. Lancet 389:519-527, 2017

© 2020 Unger JM et al. *JAMA Network Open*.
